# Symptom attributions in patients with colorectal cancer

**DOI:** 10.1186/s12875-015-0315-9

**Published:** 2015-09-03

**Authors:** Line Flytkjær Jensen, Line Hvidberg, Anette Fischer Pedersen, Peter Vedsted

**Affiliations:** Research Centre for Cancer Diagnosis in Primary Care (CaP), Research Unit for General Practice, Department of Public Health, Aarhus University, Bartholins Allé 2, 8000 Aarhus C, Denmark; Section for General Medical Practice, Department of Public Health, Aarhus University, Bartholins Allé 2, 8000 Aarhus C, Denmark

## Abstract

**Background:**

Symptoms of cancer may be interpreted differently by different patients before the diagnosis. This study investigated symptom attributions in Danish patients with colorectal cancer and the potential associations with symptom type, socio-demographic characteristics and patient interval.

**Methods:**

Data were collected among incident colorectal cancer patients (*n* = 577, response rate 64.2 %), who were asked to think back on the time before their diagnosis when completing the questionnaire. The questionnaire comprised a Danish version of the revised Illness Perception Questionnaire (IPQ-R) with questions on 19 symptom attributions. These 19 attribitutions were categorised into five causal groups for statistical analyses. The patient interval (i.e. the time from the patient’s first symptom experience to presentation to the healthcare system) was assessed in the same questionnaire. Data on socio-demographic characteristics were obtained by using nationwide registers from Statistics Denmark.

**Results:**

Patients who experienced ‘blood in stool’ as the most important symptom were more likely to attribute this to cancer (PR_ad_ 1.90, 95 % CI 1.43-2.54) and benign somatic causes (PR_ad_ 1.33, 95 % CI 1.02-1.72), such as haemorrhoids, compared to patients who did not perceive this symptom as the most important. Socio-demographic characteristics were also associated with symptom attribution. Patients with higher educational levels were less likely to attribute their most important symptom to psychological causes (PR_ad_ 0.57, 95 % CI 0.35–0.94) than patients with lower educational levels. Patients with rectal cancer attributed their most important symptom to a benign somatic cause more often than patients with colon cancer (PR_ad_ 1.39, 95 % CI 1.07–1.80).

**Conclusions:**

Symptom attribution in patients was associated with aspects of socio-demography and with the symptom type perceived by the patient as the most important. No significant associations were found between symptom attributions and patient interval. These results have implications for general practice as symptom attributions may prompt patients to present symptoms in a certain way and thereby influence the general practitioner’s assessment of presented symptoms.

## Background

Colorectal cancer (CRC) is the second most common cancer in Europe, both in terms of cancer incidence and cause of cancer death [1], and the prognosis is dependent on early diagnosis [2]. Thus, a key factor in improving the survival is the promotion of early diagnosis.

More than 98 % of the Danish population is listed with a general practitioner (GP) [3]. The GP is the first point of contact in the healthcare system and is involved in the initial diagnosis of approximately 85 % of all cancer cases [4]. Therefore, early diagnosis of CRC is highly dependent on the interaction between the patient’s interpretation and presentation of symptoms and the GP’s response to the presented symptoms [5].

The patient’s symptom interpretation depends on several factors. Firstly, the specific symptom experienced by the patient may influence the way that it is interpreted by the patient, presented to others and which attributions are given to the symptom. One study reported that a patient’s thoughts about a symptom may vary according to the nature of that particular symptom, and patients who experienced blood in stools were more likely to fear that this was a symptom of cancer [6]. Secondly, some studies have found that people of low socio-economic position (SEP) experience more difficulties with interpreting health messages and may be less aware of which symptoms to attribute to cancer than people with a higher SEP [7]. Finally, the social context and the specific situation can influence the way people attribute symptoms to different explanations [7].

Previous studies have shown that the length of the patient interval, i.e. the time from the patient experiences the first symptom until healthcare is sought [8], varies among patients [4, 9]. Aspects associated with longer patient intervals include assumption that symptoms were not serious, denial of symptoms, fear of serious illness and lower levels of education [10].

Therefore, we need more knowledge about the patients’ thoughts about the symptoms presented to their GP which later turned out to be signs of cancer. Such knowledge can be obtained by studying the patients’ symptom attributions and identifying factors associated with these attributions. This information may be useful not only in future public health campaigns, but may also impact the patient-GP encounter and the way that further symptom investigation is established.

Consequently, the aims of this study were 1) to describe CRC patients’ attributions of their most important symptom before presentation to the GP and 2) to identify associations between symptom attribution and type of symptom, socio-demographic characteristics and the patient interval.

## Methods

### Design and data collection

This study was carried out as a cross-sectional questionnaire study. All Danish citizens who had been registered with an incident, histologically confirmed CRC in the Danish Pathology Data Bank (DPDB) in the period from 1 January to 1 May 2010 were included. The DPDB is a nationwide online database in which information on all diagnostic cytological and histological specimens is registered by all Danish departments of pathology [11]. CRC patients were identified by applying the Danish version of the Systematized Nomenclature of Medicine (SNOMED) codes: T67*3 (colon), T68*3 (rectum) and T73970 (ureter and colon).

Questionnaire data were collected between 12 July and 26 August 2010, and the questionnaires were sent out 3–7 months after diagnosis. In total, 1105 patients were identified through the DPDB. Of these, 206 patients (18.6 %) were excluded because of death, unknown address or research protection (i.e. publicly recorded protection against participation in research) [12]. Thus, 899 patients received a questionnaire. Non-respondents received a reminder after three weeks, including a new copy of the questionnaire. A total of 577 questionnaires were completed and returned (response rate: 64.2 %).

The following groups of respondents were excluded from the analysis: 8.1 % (*n* = 47) reported no preceding symptoms of cancer before contacting a GP, 9.0 % (*n* = 52) had experienced several symptoms, but not indicated the most important one and 7.3 % (*n* = 42) had more than 50 % missing values in the questionnaire. In total, 48.5 % (*n* = 436) patients of the original study population were included in the statistical analyses.

### Included variables

The questionnaire contained questions on the patient interval [8]. This interval was calculated as the number of days between first symptom experience and first GP encounter. The questionnaire also comprised a Danish version of the Revised Illness Perception Questionnaire (IPQ-R) [13], including questions on symptoms and symptom attributions.

The respondents were asked to state the most important symptom they had experienced before healthcare seeking in an open-ended question with the wording: *Which of the symptoms you experienced did you perceive as the most important?* The patients were then asked to think of this symptom when answering the remaining part of the questionnaire. If the patient stated two symptoms or more as their most important symptom, the first symptom was regarded as the most important. The most important symptoms reported by the patients were categorised into the following four groups: blood in stool, abdominal pain, change in bowel habits and other symptoms. These were coded as ‘yes’ if the patient assessed the symptom as the most important and ‘no’ if not. To reduce the number of categories, some of the stated symptoms were combined. Diarrhoea and constipation were combined into *change in bowel habits*. Symptoms such as fatigue (*n* = 54), decreased appetite (*n* = 35), nausea (*n* = 19) and fever (*n* = 12) were combined into *other symptoms* since few persons stated these as the most important symptom.

The patients were also asked to state the causes they attributed to their most important symptom. Thus, in contrast to the original IPQ-R, patients were here asked to identify the perceived causes of their symptom as distinct from the causes of their illness. A more detailed description of the changes of the IPQ-R is found elsewhere [13]. The questionnaire listed 19 possible causes of the symptom; the patient was asked to rate each of these on a 5-point Likert scale. For analyses, we categorised the 19 causes into ‘agree’ (comprising the categories ‘strongly agree’ and ‘agree’) and ‘disagree’ (comprising the categories ‘strongly disagree’, ‘disagree’ and ‘neither agree nor disagree’). The 19 causes were divided into the following five groups for this study: 1. *Cancer,* 2. *Benign somatic causes* (comprising the following attributes: a germ or virus; altered immunity; already diagnosed disease; haemorrhoids; and scratch or cut), 3. *Psychological causes* (comprising the following attributes: worry; stress; my emotional state, e.g. feeling down, lonely, anxious, empty; family worry or problems; and overwork), 4. *Lifestyle-related causes* (comprising the following attributes: diet; my own behaviour; smoking; and alcohol), 5. *Causes beyond individual control* (comprising the following attributes: accident or injury; hereditary—it runs in my family; ageing; and chance or bad luck).

### Register data

These data were linked at the individual level using the unique civil registration number possessed by all Danish residents [14] and combined with information on the patients’ socio-demographic characteristics obtained from Statistics Denmark [15]. The following variables were included: age (*≤64, 65+*), gender (*male, female*), marital status *(married/cohabiting, living alone)* and highest achieved educational level (*low*: ≤10 years, *middle*: >10 ≤ 15 years and *high*: >15 years) according to UNESCO’s International Standard Classification of Education (ISCED) [16].

### Ethical considerations

The project was approved by the Danish Data Protection Agency (file no. 2009-41-3471). Because the study was based on registry and survey data, no ethical approval was required.

### Statistical analyses

The statistical analyses were performed using Stata version 13.1. Proportions were calculated to estimate the frequency of the most important symptom for patients before healthcare seeking. To study the relationship between the most important symptom and the five symptom attribution groups, we conducted generalised linear models (GLM) [17, 18] with prevalence ratios (PRs) and 95 % confidence intervals (CIs). An unadjusted model was produced and followed by a model adjusted for age, gender, marital status and education. The same method was applied to study the association between socio-demographic characteristics and the different symptom attribution groups. Furthermore, we studied attribution groups by cancer site divided into rectal and colon cancer.

The association between the five attribution groups and the patient interval was also analysed using an unadjusted GLM model and a GLM model adjusted for gender, age, marital status and education. The cut-off point for short vs long patient interval was 88 days generated from the 75th percentile from first symptom experience until healthcare seeking.

## Results

### Socio-demographic characteristics of respondents

The mean age of the respondents included in the analyses (*n* = 436) was 67.7 years; 55.7 % were males, 68.8 % were either married or cohabiting and 18.1 % had 15 or more years of education. In total, 279 were diagnosed with colon cancer, 133 with rectal cancer and 24 with both cancer types either because the patient had cancer in both colon and rectum and/or because the tumour was located in the transitional zone between the two *(data not shown)*. The distribution of socio-demographic characteristics, symptom type and cancer type is presented by the five causal groups in Table 1.

**Table 1:** Distribution of socio-demographic characteristics, cancer type, symptom type and agreement with the five symptom attribuations

	Cancer *Agree % (n)*	Total (*n*)	Benign somatic causes *Agree % (n)*	Total (*n*)	Psycho-logical causes *Agree % (n)*	Total (*n*)	Lifestyle related cause *Agree % (n)*	Total (*n*)	Causes beyond individual control *Agree % (n)*	Total (*n*)
**Gender**										
Male	33.1 (80)	242	58.6 (137)	234	**30.3 (73)**	241	33.9 (81)	239	55.5 (132)	238
Female	26.2 (50)	191	59.7 (109)	183	**42.6 (81)**	190	30.0 (56)	187	59.5 (110)	185
**Age**										
≤64	28.1 (43)	153	**68.7 (103)**	150	**41.8 (64)**	153	**39.1 (59)**	151	54.7 (82)	150
65+	31.1 (87)	280	**53.6 (143)**	267	**32.4 (90)**	278	**28.9 (78)**	275	58.6 (160)	273
**Marital status**										
Married/coh.	29.1 (87)	299	58.7 (169)	288	32.9 (98)	298	30.0 (88)	293	58.2 (171)	294
Living alone	32.1 (43)	134	59.7 (77)	129	42.1 (56)	133	36.8 (49)	133	55.0 (71)	129
**Education**										
Short	28.6 (42)	147	**46.8 (66)**	141	**43.2 (63)**	146	35.2 (51)	145	61.5 (88)	143
Middle	31.3 (62)	198	**67.0 (126)**	188	**33.5 (66)**	197	29.0 (58)	193	57.7 (112)	194
High	28.6 (22)	77	**63.6 (49)**	77	**26.0 (20)**	77	35.1 (27)	77	48.0 (36)	75
**Cancer type**										
Colon	28.6 (79)	276	**51.1 (135)**	264	37.8 (104)	275	32.8 (89)	271	58.9 (159)	270
Rectal	31.6 (42)	133	**72.1 (93)**	129	31.8 (42)	132	31.3 (41)	131	53.5 (69)	129
**Blood in stool**										
Yes	**44.1 (63)**	143	**71.9 (100)**	139	32.2 (46)	143	26.2 (37)	141	60.0 (84)	140
No	**23.1 (67)**	290	**52.5 (146)**	278	37.5 (108)	288	35.1 (100)	285	55.8 (158)	283
**Abdominal pain**										
Yes	22.9 (16)	70	**43.1 (28)**	65	41.4 (29)	70	**42.9 (30)**	70	55.9 (38)	68
No	31.4 (114)	363	**61.9 (218)**	352	34.6 (125)	361	**30.1 (107)**	356	57.5 (204)	355
**Change in bowel habits**										
Yes	26.4 (33)	125	63.4 (78)	123	36.0 (45)	125	36.3 (45)	124	55.7 (68)	122
No	31.5 (97)	308	57.1 (168)	294	35.6 (109)	306	30.5 (92)	302	57.8 (174)	301
**Other symptoms** ^a^										
Yes	**19.0 (18)**	95	**44.4 (40)**	90	36.6 (34)	93	27.5 (25)	91	55.9 (52)	93
No	**33.1 (112)**	338	**63.0 (206)**	327	35.5 (120)	338	33.4 (112)	335	57.6 (190)	330

Bold font represents statistically significant differences between groups *p* < .05 (chi2)

^a^Symptoms such as fatigue, nausea, fever and decreased appetite

### Most important symptom and attributed causes

The majority of the respondents stated blood in stool as the most important symptom (32.8 %), followed by changes in bowel habits (28.9 %), other symptom (22.0 %) and abdominal pain (16.3 %) (*data not shown*).

Table 2 presents the association between the most important symptom and the five symptom attribution groups. Respondents who stated blood in stool as the most important symptom were statistically significantly more likely to attribute this symptom to cancer (PR_ad_ 1.94, 95 % CI 1.46–2.58) or to benign somatic causes (PR_ad_ 1.36, 95 % CI 1.05-1.76) than respondents who did not report blood in stool as the most important symptom. Among respondents who reported abdominal pain as the most important symptom, statistically significantly more respondents attributed this symptom to lifestyle-related causes (PR_unad_ 1.43, 95 % CI 1.04–1.95) and statistically significantly fewer respondents attributed this symptom to benign somatic causes (PR_unad_ 0.70, 95 % CI 0.52–0.93). However, only the latter association remained statistically significant after adjustment. Respondents with other symptoms were statistically significantly more likely *not* to attribute their symptoms to cancer or to benign somatic causes. However, the latter finding was only statistically significant in the unadjusted analysis.

**Table 2:** Prevalence ratios^a^ and 95 % CI of most important symptom and the five symptom attributions

	Cancer		Benign somatic causes		Psychological causes		Lifestyle-related causes		Causes beyond individual control	
	Unadjusted	Adjusted	Unadjusted	Adjusted	Unadjusted	Adjusted	Unadjusted	Adjusted	Unadjusted	Adjusted
Blood in stool										
No	1 (ref.)	1 (ref.)	1 (ref.)	1 (ref.)	1 (ref.)	1 (ref.)	1 (ref.)	1 (ref.)	1 (ref.)	1 (ref.)
Yes	**1.91 (1.44–2.52)**	**1.94 (1.46–2.58)**	**1.37 (1.18–1.60)**	**1.36 (1.05–1.76)**	0.86 (0.65–1.14)	0.89 (0.68–1.18)	0.75 (0.54–1.03)	0.76 (0.52–1.11)	1.07 (0.91–1.27)	1.10 (0.93–1.30)
Abdominal pain										
No	1 (ref.)	1 (ref.)	1 (ref.)	1 (ref.)	1 (ref.)	1 (ref.)	1 (ref.)	1 (ref.)	1 (ref.)	1 (ref.)
Yes	0.73 (0.46–1.15)	0.76 (0.48–1.20)	**0.70 (0.52–0.93)**	0.65 (0.44–0.97)	1.20 (0.88–1.64)	1.06 (0.70–1.61)	**1.43 (1.04–1.95)**	1.26 (0.83–1.92)	0.97 (0.77–1.22)	0.97 (0.77–1.22)
Change in bowel habits										
No	1 (ref.)	1 (ref.)	1 (ref.)	1 (ref.)	1 (ref.)	1 (ref.)	1 (ref.)	1 (ref.)	1 (ref.)	1 (ref.)
Yes	0.84 (0.60–1.17)	0.80 (0.57–1.13)	1.11 (0.94–1.31)	1.10 (0.87–1.45)	1.01 (0.77–1.33)	1.02 (0.72–1.46)	1.19 (0.89–1.59)	1.24 (0.86–1.78)	0.96 (0.80–1.16)	0.96 (0.80–1.16)
Other symptoms^b^										
No	1 (ref.)	1 (ref.)	1 (ref.)	1 (ref.)	1 (ref.)	1 (ref.)	1 (ref.)	1 (ref.)	1 (ref.)	1 (ref.)
Yes	**0.57 (0.37–0.89)**	**0.54 (0.33–0.86)**	**0.71 (0.55–0.90)**	0.75 (0.53–1.05)	1.03 (0.76–1.40)	1.05 (0.71–1.56)	0.82 (0.57–1.19)	0.86 (0.54–1.35)	0.97 (0.79–1.19)	0.94 (0.76–1.16)

^*^Numbers in bold are significant results

^a^Adjusted for age, gender, marital status and education

^b^Symptoms such as fatigue, nausea, fever and decreased appetite

### Socio-demographic characteristics and causes attributed to the symptom

Respondents aged 65 years or older were found to be statistically significantly less likely than respondents aged less than 65 years to attribute their symptom to psychological causes (PR_ad_ 0.70, 95 % CI 0.50–0.98) and lifestyle-related causes (PR_ad_ 0.67, 95 % CI 0.47–0.95). Respondents with rectal cancer were more likely to believe that their symptom was due to a benign somatic cause, such as haemorrhoids, scratch or cut (PR_ad_ 1.34, 95 % CI 1.02–1.77).

Respondents with high educational levels were statistically less likely than respondents with low educational levels to attribute their symptom to psychological causes (PR_ad_ 0.57, 95 % CI 0.34–0.96). It should be noted that attributing symptoms to cancer was not statistically significantly associated with any of the socio-demographic characteristics (Table 3).

**Table 3:** Prevalence ratios^a^ and 95 % CI of socio-demographic characteristics and the five symptom attributions

	Cancer		Benign somatic causes		Psychological causes		Lifestyle-related causes		Causes beyond individual control	
	Unadjusted	Adjusted	Unadjusted	Adjusted	Unadjusted	Adjusted	Unadjusted	Adjusted	Unadjusted	Adjusted
Gender										
Male	1 (ref.)	1 (ref.)	1 (ref.)	1 (ref.)	1 (ref.)	1 (ref.)	1 (ref.)	1 (ref.)	1 (ref.)	1 (ref.)
Female	0.79 (0.59–1.07)	0.79 (0.80–1.51)	1.02 (0.87–1.19)	1.03 (0.79–1.33)	**1.41 (1.09–1.81)**	1.34 (0.95–1.84)	0.88 (0.67–1.17)	0.79 (0.56–1.13)	1.07 (0.91–1.26)	1.07 (0.82–1.39)
Age										
≤64	1 (ref.)	1 (ref.)	1 (ref.)	1 (ref.)	1 (ref.)	1 (ref.)	1 (ref.)	1 (ref.)	1 (ref.)	1 (ref.)
65+	1.11 (0.81–1.50)	1.10 (0.80–1.51)	**0.78 (0.68–0.91)**	0.82 (0.63–1.06)	**0.77 (0.60–1.00)**	**0.70 (0.50–0.98)**	**0.73 (0.55–0.95)**	**0.67 (0.47–0.95)**	1.07 (0.90–1.28)	1.04 (0.79–1.37)
Marital status										
Married/cohabiting	1 (ref.)	1 (ref.)	1 (ref.)	1 (ref.)	1 (ref.)	1 (ref.)	1 (ref.)	1 (ref.)	1 (ref.)	1 (ref.)
Living alone	1.10 (0.81–1.49)	1.16 (0.84–1.59)	1.02 (0.86–1.21)	1.05 (0.79–1.39)	1.28 (0.99–1.66)	1.17 (0.83–1.65)	1.23 (0.92–1.63)	1.27 (0.88–1.83)	0.95 (0.79–1.14)	0.90 (0.67–1.21)
Education										
Low	1 (ref.)	1 (ref.)	1 (ref.)	1 (ref.)	1 (ref.)	1 (ref.)	1 (ref.)	1 (ref.)	1 (ref.)	1 (ref.)
Middle	1.10 (0.79–1.52)	1.09 (0.78–1.52)	**1.43 (1.17–1.75)**	**1.39 (1.03–1.89)**	0.78 (0.59–1.02)	0.76 (0.54–1.09)	0.82 (0.60–1.13)	0.77 (0.52–1.13)	0.94 (0.79–1.12)	0.94 (0.70–1.25)
High	1.00 (0.65–1.55)	1.01 (0.65–1.57)	**1.36 (1.07–1.73)**	1.30 (0.89–1.90)	**0.60 (0.40–0.92)**	**0.57 (0.34–0.96)**	1.00 (0.68–1.45)	0.91 (0.56–1.47)	0.78 (0.60–1.02)	0.780 (0.53–1.16)
Cancer site										
Colon	1 (ref.)	1 (ref.)	1 (ref.)	1 (ref.)	1 (ref.)	1 (ref.)	1 (ref.)	1 (ref.)	1 (ref.)	1 (ref.)
Rectal	1.10 (0.81–1.51)	1.05 (0.76–1.46)	**1.41 (1.20–1.65)**	**1.34 (1.02–1.77)**	0.84 (0.63–1.13)	0.83 (0.57–1.21)	0.95 (0.70–1.29)	0.84 (0.58–1.24)	0.91 (0.75–1.10)	0.92 (0.68–1.24)

^*^Numbers in bold are significant results

^a^Adjusted for age, gender, marital status and education

### Symptom attributions and patient interval

Table 4 shows the association between the five symptom attribution groups and a long patient interval. There was a tendency that an attribution to a benign somatic disease was associated with longer patient intervals, but the association did not reach statistical significance (PR_ad_ 1.40, 95 % CI 0.95–2.06).

**Table 4:** Prevalence ratios^a^ and 95 % CI for association between long interval and the five symptom attributions

Prevalence ratio for having a long patient interval (i.e. ≥ 88 days)		
	Unadjusted	Adjusted
Cancer		
Disagree	1 (ref.)	1 (ref.)
Agree	0.76 (0.51–1.13)	0.77 (0.51–1.15)
Benign somatic causes		
Disagree	1 (ref.)	1 (ref.)
Agree	1.40 (0.96–2.03)	1.40 (0.95–2.06)
Psychological causes		
Disagree	1 (ref.)	1 (ref.)
Agree	1.23 (0.87–1.74)	1.25 (0.88–1.79)
Lifestyle-related causes		
Disagree	1 (ref.)	1 (ref.)
Agree	1.20 (0.84–1.71)	1.11 (0.77–1.59)
Causes beyond individual control		
Disagree	1 (ref.)	1 (ref.)
Agree	0.99 (0.70–1.41)	1.02 (0.72–1.45)

^a^Adjusted for age, gender, marital status and education

## Discussion

### Main results

The symptom ‘blood in stool’ was most often mentioned as the most important symptom. However, non-specific symptoms like fatigue, nausea, fever and decreased appetite were stated as the most important symptom by approximately one in four of all respondents. We found significant associations between the symptoms perceived as the most important and the attributions given to these symptoms. For example, patients who reported their most important symptom to be blood in stool were more likely to attribute this to cancer or benign somatic causes than patients who did not assess blood in stool as the most important symptom. Having rectal cancer was associated with a benign attribution. Patients aged 65 years or older were less likely to attribute symptoms to lifestyle or psychological explanations, and patients with lower educational levels were more likely to attribute their symptom to psychological causes. A long patient interval tended to be associated with attribution to benign explanations, although this propensity was not statistically significant.

### Discussion of results

In line with another study [19], we found that a non-specific symptom is commonly seen as one of the first signs among cancer patients. Studies indicate that non-specific symptoms like fatigue and weight loss are harder for patients to interpret than specific alarm symptoms like a lump in the breast [20, 21]. This difference is also supported by this study showing that patients with the more non-specific symptoms (classified into the category ‘other symptoms’) were more likely *not* to attribute these symptoms to cancer.

Experiencing blood in stool as the most important symptom before seeking medical advice was associated with attributing the symptom to cancer in this study, which was also found in another Danish study [6]. However, we also found that statistically significantly more patients with blood in stool attributed this to benign somatic causes (e.g. haemorrhoid, scratch or cut). This finding is in line with the results of another study, which found that the majority of cancer patients initially attribute their symptoms to other illnesses than cancer; more than 50 % attribute their symptoms to haemorrhoids [22]. Blood in stool is a common symptom [23] which is often causen by e.g. haemorrhoids. Thus, attributing blood in stool to haemorrhoids may be common sense. Hence, a review on symptom representations found that attributing cancer symptoms to other ailments than cancer is common among cancer patients [24]. These results indicate that some cancer patients did attribute their initial symptoms to cancer, but other patients attribute their symptoms to something else; this applies both to patients with specific and non-specific symptoms.

The crucial challenge for patients is to distinguish between benign and malignant symptoms [24]. Several studies have found that many people are not aware of the symptoms associated with colorectal cancer [25–27]. However, the implications for public health campaigns are complex. On the one hand, it is an important message for the population that symptoms such as blood in stool and more non-specific symptoms like persistent fatigue and weight loss could be due to cancer. On the other hand, fear of cancer has also shown to increase the risk of long patient intervals [28]. Moreover, GPs would probably not be able to accommodate the extra workload if every person with rectal bleeding and fatigue immediately presented these symptoms to their GP.

It is well established that people with a higher educational level and higher income are more likely to have better health outcomes [29, 30]. It has also previously been found that people with a high SEP are more likely to seek medical help faster than people with a low SEP [31, 32]. This study also indicates that symptom attributions among cancer patients before diagnosis vary between socio-demographic groups. For example, patients with lower educational levels were more likely to attribute their symptom to psychological causes than patients with higher educational levels, whereas patients aged 65 years or older were less likely to attribute their symptom to psychological and lifestyle-related causes than patients aged 64 years or younger. This variation across socio-demographic groups may be valuable knowledge for GPs because patients bring their own interpretations of their symptoms when these are presented to a medical professional. Our study indicates that among patients who later got CRC, the GP can meet patients who are ‘normalising’ their symptom and attributing it to a benign explanation when presenting their symptom in the medical consultation. This is a valuable insight, which must be kept in mind in the clinical encounter.

No statistically significant associations were observed between symptom attributions and the patient interval. Still, there was a tendency that attribution to benign disease leads to longer patient intervals.

### Discussion of methods

To the best of our knowledge, this study is the first to deploy a theoretical framework (i.e. Leventhal’s Common Sense model and the IPQ-R [33]) to examine symptom attributions before a CRC diagnosis. We consider this a key strength. Various data sources were used in this study, and the application of the unique Danish civil registration number [14] enabled us to link all data sources. The CRC patients were identified through the DPDB, which has previously been validated and found to be a reliable tool [11]. However, registrations prior to 1997 were incomplete. This imples that some recurrent cancers might be present, and it is likely that the symptom attribution and healthcare-seeking behaviour of recurrent cancer patients is distinct from that of incident cancer patients. Variables on socio-demographic characteristics were obtained from national registers containing valid individual-specific information [34]. Our response rate of 64.2 % is higher than in comparable studies for this population (i.e. diagnosed cancer patients) [6, 35]. Moreover, from a previous study on this population, we know that non-respondents tend to be significantly older than respondents [13].

The information about the patients’ most important symptom, symptom attributions and patient interval was obtained by a questionnaire, and the appropriateness of this method could be discussed. Each patient was asked to state the most important symptom experienced prior to help-seeking and to think of this symptom when answering the IPQ-R. This was done as patients with several symptoms may have had different interpretations to each of the symptoms making the questionnaire impossible to complete. This has two important implications. Firstly, the reference group used for comparison with each of the four symptom groups comprises patients who did not assess the symptom as the most important, i.e. the symptom may have been experienced although not assessed as the most important one. This was e.g. the case for 21 % of the patients who did not assess their most important symptom to be blood in stool, but they still reported to have experienced this symptom. Secondly, although we intended to study the most important symptom as perceived by the patient, several other symptoms might also have been experienced by the patient. These symptoms may have interacted and created different reactions in combination (e.g. patients with both blood in stool and changes in bowel habits may think differently about their symptoms than patients with fatigue and changes in bowel habits). Future studies should examine more thoroughly the complex interactions between frequently occurring symptom combinations and attributed causes.

Another crucial point is that we aimed to study symptom attributions prior to diagnosis among a group of already diagnosed patients. Hence, we relied on the patients’ ability to distinguish between their symptom experiences before and after becoming a cancer patient. As patients will often try to rationalise and legitimate their decision to seek care [36], a retrospective design may introduce recall bias as also pointed out by several former studies [37, 38]. However, other study designs may also introduce methodological challenges. It would be unfeasible to follow a large population in a prospective design and continuously study their symptom experiences in detail because only very few will develop cancer and thus be eligible for study. Thus, although the applied design has limitations, it may be the only feasible method. Several points indicate that the patients were not affected by the retrospective assessments of symptoms and help-seeking behaviour. Firstly, we validated the psychometric properties of symptom attribution as a construct in this patient group [13]. This validation indicated that a small proportion of patients were thinking of their disease instead of their symptom when answering the questionnaire [13]. We also found that experiencing the alarm symptom ‘blood in stool’ as the most important symptom was associated with both attributing this to cancer and to a benign somatic cause. This could indicate that the patients did not answer in one direction because they were now aware that the symptoms were due to cancer.

Among our study population, 279 were diagnosed with colon cancer, 133 with rectal cancer and 24 with both cancer types. This distribution reflects the overall incidence in Denmark, where about two thirds of all colorectal tumours develop in the colon [39]. Patients with colon cancer often present with vague symptoms such as weight loss and fatigue, whereas cancer located in the rectum often causes symptoms like abnormal defecation with blood or mucus [40]. As patients with these cancer types seem to experience different symptoms, at least to some degree, this may have affected our results because symptom attributions are associated with the most important symptom experienced.

## Conclusions

This study provided insights into CRC patients’ attributions of their most important symptom prior to their diagnosis and into factors associated with these attributions. The results indicate that patients who assessed their most important symptom to be blood in stool were more likely to attribute this to cancer or to a benign somatic cause and that patients with low educational levels were more likely to attribute their symptom to psychological causes. This is important information as false symptom interpretations may impact the patient-GP encounter and may have later implications for the timespan from symptom presentation to referral for further investigations.

However, more research is needed in this area. It could be valuable to examine the consequences of self-labelling (i.e. the patient tells the GP what they thinks is the most likely diagnosis, which may or may not be correct) [41] for the clinical decision-making, for the time spent in primary care and ultimately for the diagnosis of CRC.

## Abbreviations

CI: Confidence interval
CRC: Colorectal cancer
DPDB: Danish Pathology Data Bank
GLM: Generalised linear models
GP: General practitioner
IPQ-R: Revised Illness Perception Questionnaire
ISCED: International Standard Classification of Education
PR: Prevalence ratio
PR_ad_: Adjusted prevalence ratio
PR_unad_: Unadjusted prevalence ratio
SEP: Socio-economic position
SNOMED: Systematized Nomenclature of Medicine
UNESCO: United Nations Educational, Scientific and Cultural Organization
