# Erratum to: Symptom attributions in patients with colorectal cancer

**DOI:** 10.1186/s12875-015-0338-2

**Published:** 2015-10-05

**Authors:** Line Flytkjær Jensen, Line Hvidberg, Anette Fischer Pedersen, Peter Vedsted

**Affiliations:** 1 Research Centre for Cancer Diagnosis in Primary Care (CaP), Research Unit for General Practice, Department of Public Health, Aarhus University, Bartholins Allé 2, Aarhus C, 8000 Denmark; 2 Section for General Medical Practice, Department of Public Health, Aarhus University, Bartholins Allé 2, Aarhus C, 8000 Denmark

Unfortunately, the original version of this article [1] contained an error. The original article was published containing the following paragraph in the abstract “Patients who experienced ‘blood in stool’ as the most important symptom were more likely to attribute this to cancer (PR _ad_ 1.90, 95 % CI 1.43–2.54) and benign somatic causes (PR _ad_ 1.33, 95 % CI 1.02–1.72), such as haemorrhoids, compared to patients who did not perceive this symptom as the most important. Socio-demographic characteristics were also associated with symptom attribution. Patients with higher educational levels were less likely to attribute their most important symptom to psychological causes (PR _ad_ 0.57, 95 % CI 0.35–0.94) than patients with lower educational levels. Patients with rectal cancer attributed their most important symptom to a benign somatic cause more often than patients with colon cancer (PR _ad_ 1.39, 95 % CI 1.07–1.80).”

This should have read “Patients who experienced ‘blood in stool’ as the most important symptom were more likely to attribute this to cancer (PR _ad_ 1.94, 95 % CI 1.46-2.58) and benign somatic causes (PR _ad_ 1.36, 95 % CI 1.05–1.76), such as haemorrhoids, compared to patients who did not perceive this symptom as the most important. Socio-demographic characteristics were also associated with symptom attribution. Patients with higher educational levels were less likely to attribute their most important symptom to psychological causes (PR _ad_ 0.57, 95 % CI 0.34–0.96) than patients with lower educational levels. Patients with rectal cancer attributed their most important symptom to a benign somatic cause more often than patients with colon cancer (PR _ad_ 1.34, 95 % CI 1.02–1.77).”

This has now been corrected in the original article.
